# DNA Topology and the Initiation of Virus DNA Packaging

**DOI:** 10.1371/journal.pone.0154785

**Published:** 2016-05-04

**Authors:** Choon Seok Oh, Jean Sippy, Bridget Charbonneau, Jennifer Crow Hutchinson, Olga Esther Mejia-Romero, Michael Barton, Priyal Patel, Rachel Sippy, Michael Feiss

**Affiliations:** Department of Microbiology, University of Iowa, Iowa City, Iowa, United States of America; University of Oklahoma, UNITED STATES

## Abstract

During progeny assembly, viruses selectively package virion genomes from a nucleic acid pool that includes host nucleic acids. For large dsDNA viruses, including tailed bacteriophages and herpesviruses, immature viral DNA is recognized and translocated into a preformed icosahedral shell, the prohead. Recognition involves specific interactions between the viral packaging enzyme, terminase, and viral DNA recognition sites. Generally, viral DNA is recognized by terminase’s small subunit (TerS). The large terminase subunit (TerL) contains translocation ATPase and endonuclease domains. In phage lambda, TerS binds a sequence repeated three times in *cosB*, the recognition site. TerS binding to *cosB* positions TerL to cut the concatemeric DNA at the adjacent nicking site, *cosN*. TerL introduces staggered nicks in *cosN*, generating twelve bp cohesive ends. Terminase separates the cohesive ends and remains bound to the *cosB*-containing end, in a nucleoprotein structure called Complex I. Complex I docks on the prohead’s portal vertex and translocation ensues. DNA topology plays a role in the TerS^λ^-*cosB*^*λ*^ interaction. Here we show that a site, *I2*, located between *cosN* and *cosB*, is critically important for an early DNA packaging step. *I2* contains a complex static bend. *I2* mutations block DNA packaging. *I2* mutant DNA is cut by terminase at *cosN in vitro*, but *in vivo*, no *cos* cleavage is detected, nor is there evidence for Complex I. Models for what packaging step might be blocked by *I2* mutations are presented.

## Introduction

Large dsDNA viruses use an ATP hydrolysis-powered motor to package DNA into preformed empty shells, called proheads (reviewed in [1–4]). For many tailed bacteriophages and the herpesviruses, replication and recombination produce concatemers, i.e., end-to-end multimers of viral chromosomes. During packaging, an endonucleolytic cut is made to generate the DNA end which is translocated into the prohead to initiate DNA packaging. After prohead filling, a second DNA cut terminates translocation. The endonucleolytic cuts and translocation are sponsored by a multifunctional viral enzyme, terminase. Terminases are generally hetero-oligomers of large (TerL) and small (TerS) subunits. TerL contains the translocation ATPase in an N-terminal domain, and the concatemer-processing endonuclease in a C-terminal domain. Many phages, including P22, Sf6, SPP1, and T4 use a headful packaging strategy, in which the initial cut is specific, but subsequent, non-specific cuts are triggered when the prohead is full [5–7]. Terminases act processively, such that after the downstream cut, terminase remains bound to the newly-created end in a complex that then binds a naive prohead, and sponsors packaging of the next chromosome along the concatemer.

**Fig 1:**
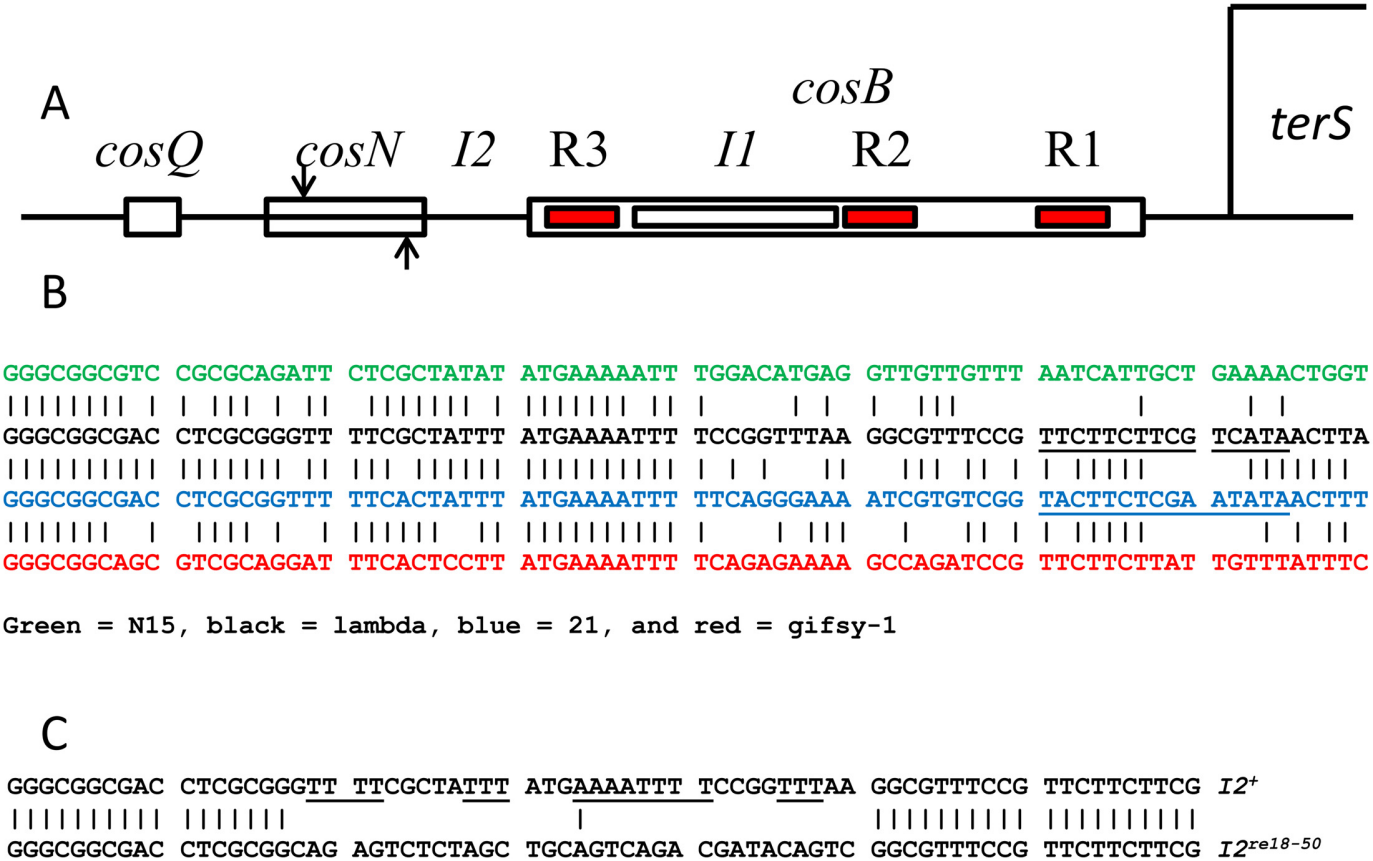
Elements of *cos*. A. Structure of *cos*^*λ*^. *cosN* is the site at which TerL endonuclease centers introduce staggered nicks to generate the cohesive ends of λ virion DNA. *cosB* is the complex site at which TerS binds to anchor TerL: R3, R2 and R1 are TerS binding sites, and *I1* is a binding site for the *E*. *coli* site-specific DNA bending protein, IHF. *I2* is located between *cosN* and *cosB*. *cosQ* is essential for DNA packaging termination. B. Alignment of the *I2*-containing left DNA ends, i.e., bp 1–70, of λ-like phages N15 (green), λ (black), 21 (blue) and gifsy-1 (red). The *I2* segment extends approximately from bp 18 to 50. Approximate positions of R3 segments are underlined. C. The sequence of the left DNA end, bp 1–70, of *I2*^+^ (above) and *I2*^*re18-50*^ (below). The *I2*^*re18-50*^ mutation replaces the AT-rich *I2*^+^ sequence without changing the *cosN-cosB* spacing. Underlining highlights the poly-dA and poly-dT segments of *I2*^+^.

In contrast to the headful strategy, many virion DNAs, including those of λ-like phages λ, 434, 21, Φ80, N15, and gifsy-1 have cohesive ends: 12 base-long, complementary 5’ single stranded extensions that are generated, during DNA packaging, when terminase introduces staggered nicks at the *cos* sites of concatemers [8]. λ’s *cos* (*cos*^*λ*^) contains three sub-sites whose interactions with the packaging machinery orchestrate the recognition, cleavage and packaging of viral chromosomes, as follows [8, 9]. *cosN* is an *ca*. 22 bp-long site at which terminase introduces staggered nicks to create the cohesive ends of virion DNA molecules. Initiation of DNA packaging requires *cosN* and the adjacent sub-site, *cosB*. *cosB*^*λ*^ is complex, consisting of three binding sites, R3, R2, and R1, for TerS (Fig 1A). TerS^λ^’s N-terminus contains the DNA binding domain (DBD), a winged helix-turn-helix (wHTH) DNA binding motif that interacts with the *cosB* R sequences [10, 11]. The N-terminal DBD of TerS^λ^, along with that of phage N15, a λ-like phage, forms a tight dimer [10, 12]. In contrast, the DBD domains of *pac* phages Sf6, P22, and SF6 are monomeric [13–15]. The recognition helixes of TerS^λ^’s dimeric wHTH motifs are solvent-exposed and positioned appropriately for DNA binding. The terminase protomer is a TerS^λ^_2_:TerL^λ^_1_ heterotrimer [16]; the heterotrimers further oligomerize into tetramers [17], indicating that TerS octamerizes. Between R3 and R2 is *I1*, a binding site for IHF, the *E*. *coli* DNA binding and bending protein [18–22]. *I1* contains a modest, *ca*. 35°, intrinsic bend [23], and IHF binding increases the bend to ~120° [21, 22, 24]. The sharp IHF-enhanced bend at *I1* positions the major grooves of R3 and R2 to face each other, creating a structure into which the dimeric TerS^λ^ DNA binding domain can dock [10]. When *cosB* is deleted, or the *cosB-cosN* spacing is altered, terminase nicking of *cosN* is inaccurate, indicating that TerS^λ^ interactions with *cosB* anchor TerL^λ^, positioning the endonuclease domains on *cosN* for accurate nicking [25, 26].

Following *cosN* nicking, cohesive ends are separated, and terminase forms a tight, stable complex, Complex I, on the *cosB*-containing DNA end. Complex I protects the chromosome end from digestion by host cell nucleases, but the right, *cosQ*-containing end, is subject to nuclease attack. Complex I docks on the portal protein of a prohead and ATP hydrolysis-powered translocation of the DNA through the portal vertex into the prohead shell ensues. When the translocating complex encounters the downstream *cos*, *cosN* is nicked to complete packaging, and terminase undocks from the portal vertex and remains bound to the next chromosome, forming a new Complex I that docks on a new prohead’s portal and sponsors packaging of the downstream chromosome. Recognition of the downstream *cos* requires *cosN* and the third *cos* sub-site, *cosQ* [9, 27] An assembly chaperon, gpFI, assists in the association of Complex I with the prohead [28–32]. It is proposed that gpFI, which is bound to the prohead’s gpE lattice, acts through non-specific DNA binding, to assist in formation of the ternary complex of DNA, terminase, and prohead that leads to motor assembly and translocation [31].

Are the dramatic events at initiation of packaging accompanied by major changes in terminase organization? TerS_2_:TerL_1_ protomers, in the presence of IHF, are active in *cos* cleavage and DNA packaging [17]. The tetrameric form is competent to (1) cut *cos*, and when provided with proheads, (2) sponsor DNA packaging [17]. IHF is not required by the tetramer. These observations suggest a model in which IHF and interactions with *cosB* facilitate tetramer formation, and that the tetramer is the active form of terminase throughout the early steps of DNA packaging. This view can be reconciled with *in vivo* results, as follows. Although λ forms plaques on cells lacking IHF, the virus yield is reduced to about 30% the yield in IHF^+^ cells [33]. One possibility is that the part of λ DNA packaging that is IHF-dependent may be the fraction of tetramers that require IHF and DNA to assemble, with the remaining, IHF-independent packaging reflecting the level of tetrameric terminase assembled independently of IHF and *cos*. These models suggest that the terminase tetramer does not undergo major structural changes during the early packaging steps.

*I2* is a *ca*. 33 bp segment of unknown function between *cosN* and *cosB* (Fig 1B). An early study showed a correlation between reduction in virus yield and the size of small insertion and deletion mutations in *I2*, suggesting that the *cosN-cosB* spacing is crucial for *cos* function. The spacing changes affected initiation, but not termination, of DNA packaging. Both 7 bp and 11 bp deletion mutations were lethal [34]. Later work showed that altering the *cosN-cosB* spacing resulted in incorrect nicking, with displacement of nick positions to the right for insertions and to the left for deletions, indicating that proper *cosN-cosB* spacing positions the TerL endonuclease domains on *cosN* [25]. A 22-bp deletion of *cosN*, called *cos2*, abolishes nicking. *I2* is strongly conserved in the λ-like phages suggesting that *I2* functions as more than a spacer (Fig 1B). To study this, we investigated mutations that change the *I2* sequence but not the *cosN-cosB* spacing.

## Results

### *I2* is essential

To ask if the sequence between *cosN* and R3 is required for DNA packaging, we constructed a cosmid with an *I2* substitution mutation, *I2*^*re18-50*^, in which the 33 bp from λ bp 18 to 50 were changed. In *I2*^*re18-50*^, the *cosN-cosB* spacing was retained, but not the G+C content (Fig 1C). Isogenic cosmids differing only at *I2*, being *I2*^+^ [pBUC8] or *I2*^*re18-50*^ [pBC2] were subjected to *in vivo* cosmid packaging by a λ helper phage. In this assay, replication generates cosmid concatemers that are packaged by the helper phage DNA packaging machinery. Phages carrying linear cosmid multimers are assayed as ampicillin-transducing phages. While the *I2*^+^ cosmid gave a yield of 1.7 x 10^7^ Ap-transducing phages/ml, the *I2*^*re18-50*^ cosmid yield was <10^1^ Ap-transducing phages/ml, indicating that scrambling λ bp 18 to 50 causes a profound DNA packaging defect. The presence of the *I2*^+^ and *I2*^*re18-50*^ plasmids did not affect the helper phage yield, indicating that *I2*^*re18-50*^ acts in *cis*, at least in a plasmid background.

### *I2*^*re18-50*^ is *cis*-specific

As part of *cos*, *I2* mutations are expected to act in *cis*, i.e., to affect packaging of an *I2* mutant chromosome, but not to act in *trans*. We did a phage complementation experiment to ask if *I2*^*re18-50*^ is *cis*-specific. We first crossed the *I2*^*re18-50*^ mutation into λ-P1. We used λ-P1 as wild type λ because λ-P1’s prophage is a plasmid, making it useful for studies with lethal mutations such as *I2*^*re18-50*^ (see Materials and Methods). λ-P1 also transduces kanamycin resistance, so that the yield of a non-plaque forming derivative, i.e., λ-P1 *I2*^*re18-50*^, can be determined. As the *I2*^+^ phage, we used λ *cI857 red3*, an *att*^+^ phage that integrates its prophage into the bacterial chromosome. Lysogens of the *I2*^+^ and *I2*^*re18-50*^ phages, and a dilysogen carrying both, were constructed using the *recA*^-^ host MF3510. As expected from the above cosmid packaging experiment, *I2*^*re18-50*^ reduced the yield of λ-P1 by about 5 orders of magnitude (Table 1, compare lines 2 and 4). Because *I2*^*re18-50*^ is lethal, the yield of λ-P1 *I2*^*re18-50*^ was determined by titering kanamycin-transducing phages. The efficiency of the kanamycin-transducing phage assay was about 50% when compared with the assay for plaque-forming phages (line 4). In the lysate of the *I2*^+^ and *I2*^*re18-50*^ dilysogen, the presence of λ-P1 *I2*^*re18-50*^ resulted in a modest reduction (5-fold) in the yield of λ *I2*^+^ (Table 1, compare the plaque forming phage yields in lines 1 and 3). In turn, the profoundly low yield of λ *I2*^*re18-50*^ from a mono-lysogen was increased slightly (30-fold) by λ *I2*^+^, but remained severely low (compare the kanamycin transducing phage yields in lines 2 and 3). We ascribe the modest increase in the yield of λ-P1 *I2*^*re18-50*^ to the effect of λ *I2*^+^ on increased concatemer production and late gene dosage (see Materials and Methods). The yield of the λ *I2*^*re18-50*^ phage is >2000-fold less than that of λ *I2*^+^, confirming that the *I2*^*re18-50*^ defect is *cis*-specific (line 3, compare the plaque forming phage yield of λ *I2*^+^ with the kanamycin transducing phage yield of λ-P1 *I2*^*re18-50*^). As a control, we looked at the complementation behavior of a phage carrying a mutation, *Aam42*, expected to be recessive to the wild type *A*^+^ allele. *Aam42* is a nonsense mutation of the fifth-to-last codon of the *A* gene [35]. The truncated gpA of λ-P1 *Aam42* lacks the C-terminus of the prohead binding domain, and the resulting terminase is defective in prohead binding. We reasoned that in a coinfection by λ *A*^+^ and λ-P1 *Aam42*, wild type terminase would act in *trans*, sponsoring packaging of both *A*^+^ and *Aam42* DNAs. Induction of a mono-lysogen of λ-P1 *Aam42* alone resulted in a very low virus yield (line 6), as expected for an amber mutant. Coinfection with λ *A*^+^ increased the yield of λ-P1 *Aam42* about 2000-fold (line 7), indicating that the *Aam42* mutation indeed is recessive.

**Table 1:** The *I2*^*re18-50*^ mutation is *cis*-specific.

Line	Prophage(s)	Yield of plaque forming units / induced lysogen	Yield of kanamycin-transducing phages / induced lysogen
1	λ *cI857 red3*	**33.9**	**-**
2	λ-P1 *I2*^*re18-50*^	**<5 x 10**^**−6**^	**6 x 10**^**−5**^
3	λ *cI857 red3*; λ-P1 *I2*^*re18-50*^	**6.35**	**1.75 x 10**^**−3**^
4	λ-P1	**11.35**	**5.65**
5	λ *cI857 red3*; λ-P1	**40.35**	**6.35**
6	λ-P1 *Aam42*	**7.3 x 10**^**−3**^	**4.1 x 10**^**−3**^
7	λ *cI857 red3*; λ-P1 *Aam42*	**21.5**	**9.3**

Host = MF3510 [*recA*1 and *sup*°]. Plaque forming phages were titered on C600; kanamycin transducing phages were titered with C600(λ^+^) as the recipient. A repeat experiment gave equivalent results.

### Location of the critical *I2* segment

**Fig 2:**
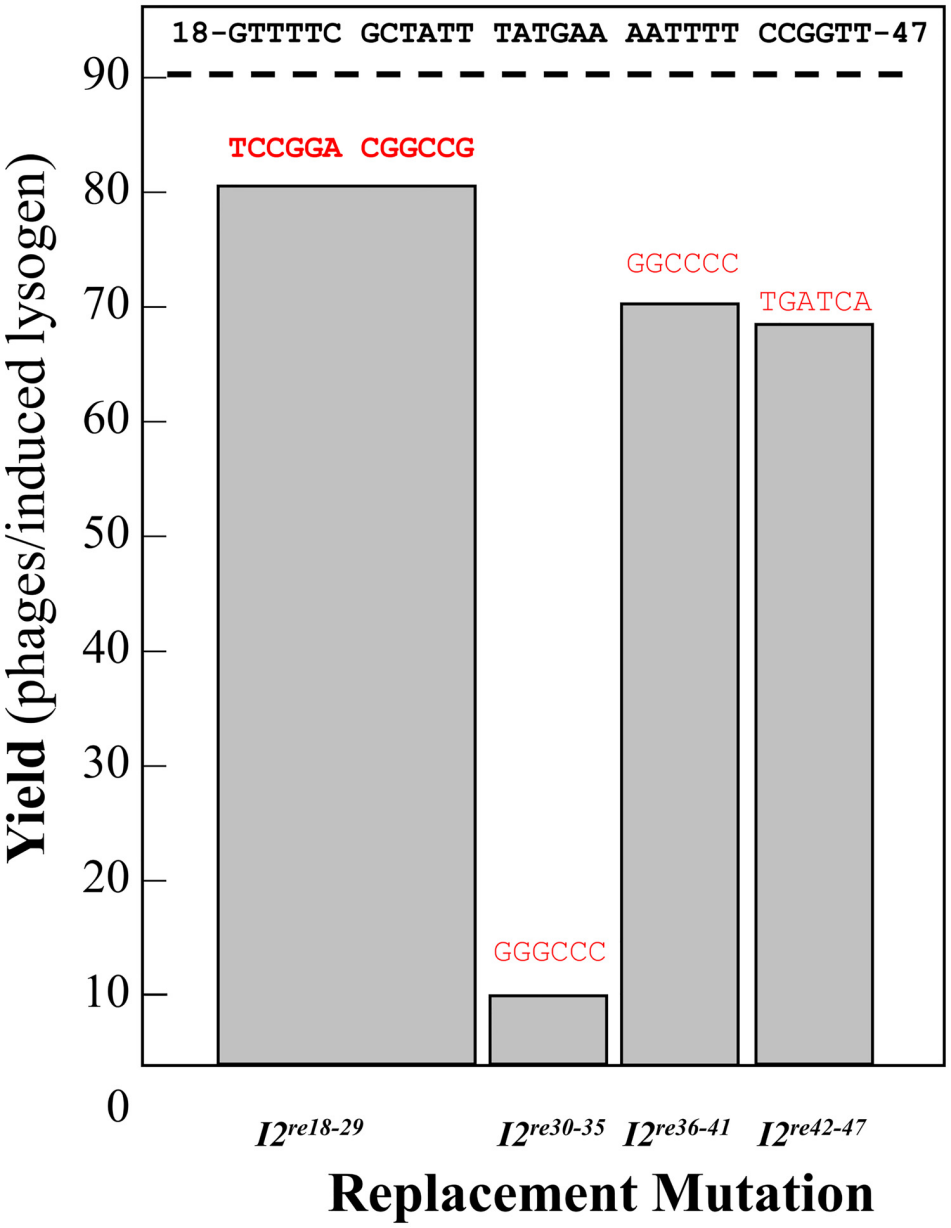
Effect of *I2*^re^ mutations on virus yield. Lysogens of λ-P1 *I2*^+^ and the *I2*^*re*^ mutants were induced and the phage yields in the resulting lysates were determined. The *I2*^+^ sequence is shown at the top of the figure in black. The sequences of the replacement mutations are in red. For the lethal *I2*^*re30-35*^ mutant, the kanamycin-transducing titer was determined. The large *I2*^*re18-29*^ mutation was used because an earlier study with 6 bp-long mutations indicated that the entire segment could be replaced without affecting virus growth. These data are from a single experiment; a repeat experiment gave equivalent results.

Scanning mutagenesis was done across *I2* by constructing a series of mutations changing blocks of 6 or 12 bp in *I2*. The mutational changes (Fig 2) were constructed in cosmids. Preliminary cosmid packaging experiments showed that a critical segment of *I2* was located in the λ bp 30–35 segment. To confirm this finding, the *I2* mutation-containing cosmids were crossed with λ-P1 *cos2*, a mutant deleted for *cosN*. Since *cosN* and *I2* are adjacent, it was expected that many of the recombinants that rescued *cosN*^+^ would also co-rescue the *I2* mutations. For most of these crosses, plaque-forming recombinants carrying the *I2* mutations were readily obtained, with the exceptions of *I2*^*re30-35*^. For *I2*^*re30-35*^, the cross lysate was used to transduce MF1427 to Kn^R^, and transductants were screened for lysogens unable to produce plaque-forming phages. These recombinant prophages were found by sequencing to be *I2*^*re30-35*^ mutants, confirming that *I2*^*re30-35*^ is lethal. Virus yield studies showed that all of the *I2* segments, except for bp 30–35, could be replaced without major effect on virus yield. In contrast, the yield of λ *I2*^*re30-35*^ was reduced to ~4 phage/cell, a level below that required for plaque formation (Fig 2). The results indicate the segment from bp 30–35 is crucial for virus viability. The much greater packaging defect of λ-P1 *I2*^*re18-50*^ (Table 1), compared to *I2*^*re30-35*^ (Fig 2), indicates that bp outside of the bp 30–35 segment play a role in *I2*’s DNA packaging function.

### A complex static bend at *I2*

**Fig 3:**
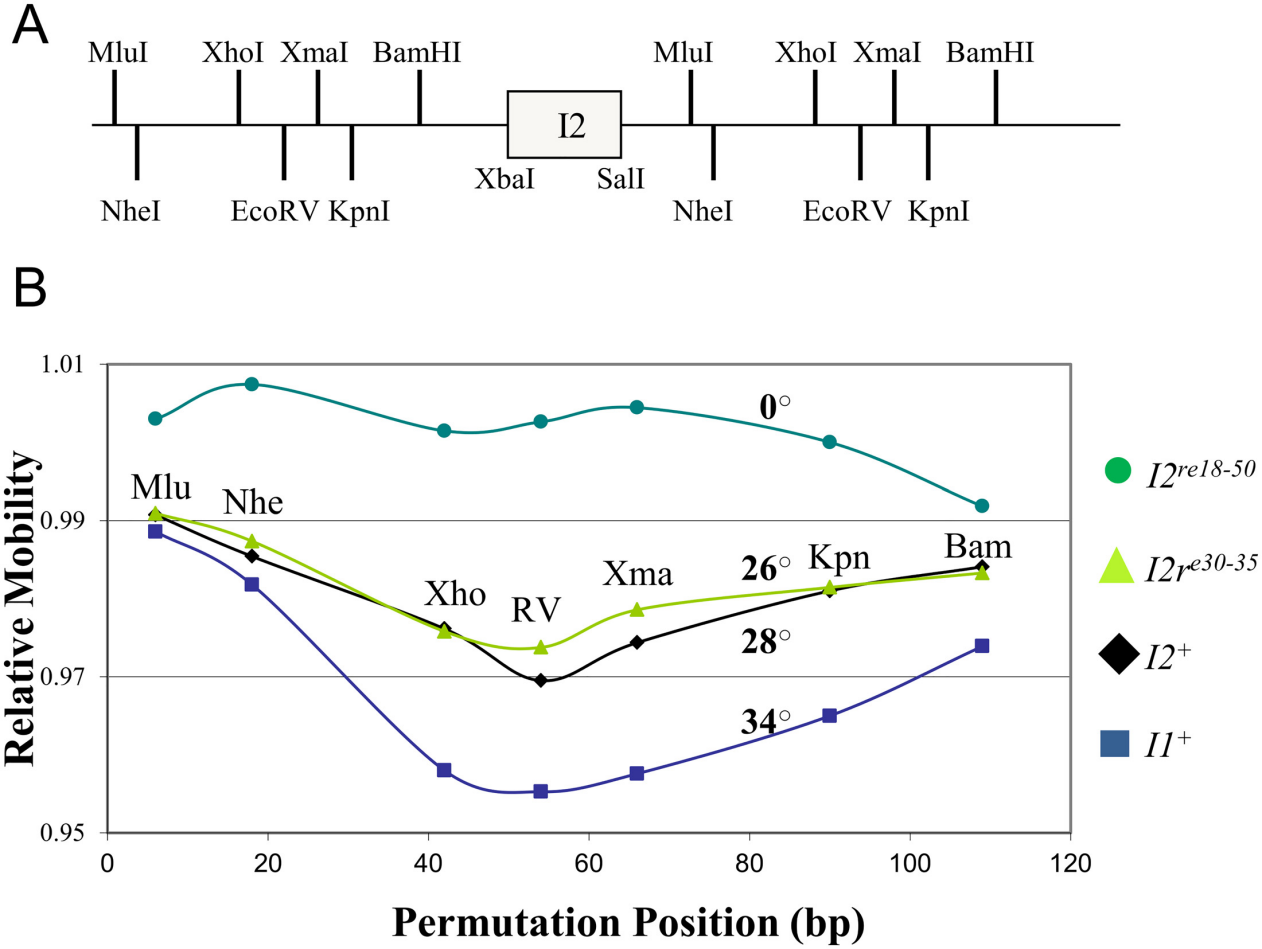
A static bend at *I2*: permutation analysis of a 150 bp DNA fragment containing λ *I1*^+^, *I2*^+^, *I2*^*re30-35*^, or *I2*^*re18-50*^. A. Diagram of the pBend plasmid showing the XbaI and SalI sites used to insert the *I2* segments. Flanking the *I2* inserts are repeated segments with the restriction enzyme target sites used to generate DNAs with permutations of *I2* position. B. Relative mobilities of the permuted DNA fragments versus positions of the *I2* segment. Relative mobility 1.0 indicates the mobility of a 150 bp DNA marker. The calculated bend angles [40] are given adjacent to the relevant mobility curve.

Poly-dA tracts centered at approximately 10–11 bp intervals cause DNA to form a static two-dimensional bend (reviewed in [36]). Inspection of the bp 18-to-50 segment indicated the presence of poly-dA tracts that were irregularly placed on both strands (underlined in Fig 1C). The *I2* poly-dA tracts suggested that there was likely a complex, i.e., non-planar, intrinsic bend at *I2*. To ask about the presence of intrinsic bending at *I2*, the electrophoretic mobility of permuted 150 bp fragments containing a 35 bp *I2* DNA insert (λ bp 18–52) was examined. As a positive control, the *I1* segment extending from λ bp 65–90 was inserted in pBend [37]. As described earlier, *I1* contains an intrinsic bend. Mobility was reduced for fragments carrying either *I1* or *I2* near the mid-point, indicating the presence of static bends in both (Fig 3). Calculations indicate the overall *I2* bend angle is about 28°, and that of the *I1* control was about 34°. The *I2*^*re30-35*^ mutation had at most a minor effect on mobility and the calculated bend angle, but the severe *I2*^*re18-50*^ mutation abolished the static bend. These results are consistent with a model in which a complex, three-dimensional bend at *I2* is required for DNA packaging.

The electrophoretic mobility data (Fig 3) indicate that *I2*^*re30-35*^ has little effect on I2’s bend, whereas the more extensive *I2*^*re18-50*^ abolishes the bend. *I2*^*re30-35*^ has little effect on apparent bending and is significantly less severe than *I2*^*re18-50*^. The lack of a change in apparent bending indicates that the bp 30–35 segment does not contribute significantly to the bending detected in the electrophoresis assay. *I2*^*re18-50*^ abolishes bending. Taken together, the results suggest that *I2* bending is produced in the *I2* bp that flank the bp 30–35 segment, i.e., upstream bp 18–29 and downstream bp 36–50. How might these two flanking segments contribute to *I2* bending? Stretches of poly-A base pairs result in DNA curvature [36, 38, 39]. Clues to bending at *I2* and the effects of the *I2* mutation come from examining the sequence. The wild type *I2* sequence is:

20. 30. 40. 50.

5’G**TTTT**CGCTATTTATG**AA****AATTTT**CCGGTTTAA-3’

3’C**AAAA**GCGATAAATAC**TT****TTAAAA**GGCCAAATT-5’

where A_4_ tracts are in bold and the bp changed by the *I2*^*re30-35*^ mutation are underlined. Note the bottom strand A_4_T_4_ and A_4_ segments, which contribute static bends, as follows. Koo and Crothers studied static bending of a very similar sequence with A_5_ and A_4_T_4_ segments. When the centers of these two segments were separated by *ca*. 10 bp, i.e., approximately a helical turn, the bends were reinforcing, producing maximal retarded gel mobility. When these segments were separated by 15 bp, i. e., out of phase, minimal gel mobility effects were observed. In the *I2* sequence, the center-to-center distance from the A_4_T_4_ sequence to the A_4_ sequence is an out-of-phase 17 bp. As the 17 bp spacing is not 1.5 turns, rather than cancelling, the two bends generate a skewed, 3-dimensional trajectory. *I2*^*re30-35*^ alters the T_4_ tract of the A_4_T_4_ sequence, leaving A_4_ tracts with a 19 bp center-to-center spacing, again a spacing neither in-phase or cancelling. Whereas the severe *I2*^*re18-50*^ change abolishes bending, the less severe *I2*^*re30-35*^ likely alters the geometry of the bending. Making the assumption that *I2*^*re30-35*^ simply eliminates the A_4_ tracts known to contribute to bending, we propose that *I2*^*re30-35*^ alters *I2* bending to produce a DNA trajectory that is unfavorable for interaction with terminase.

One alternative explanation, which we have not investigated, is that the bp 30–35 segment is flexible and permits bending at *I2* that is required for proper terminase contacts. Obviously a molecular explanation for the role of *I2* requires additional biophysical and structural studies beyond the scope of this report.

### Failure to package DNA

**Fig 4:**
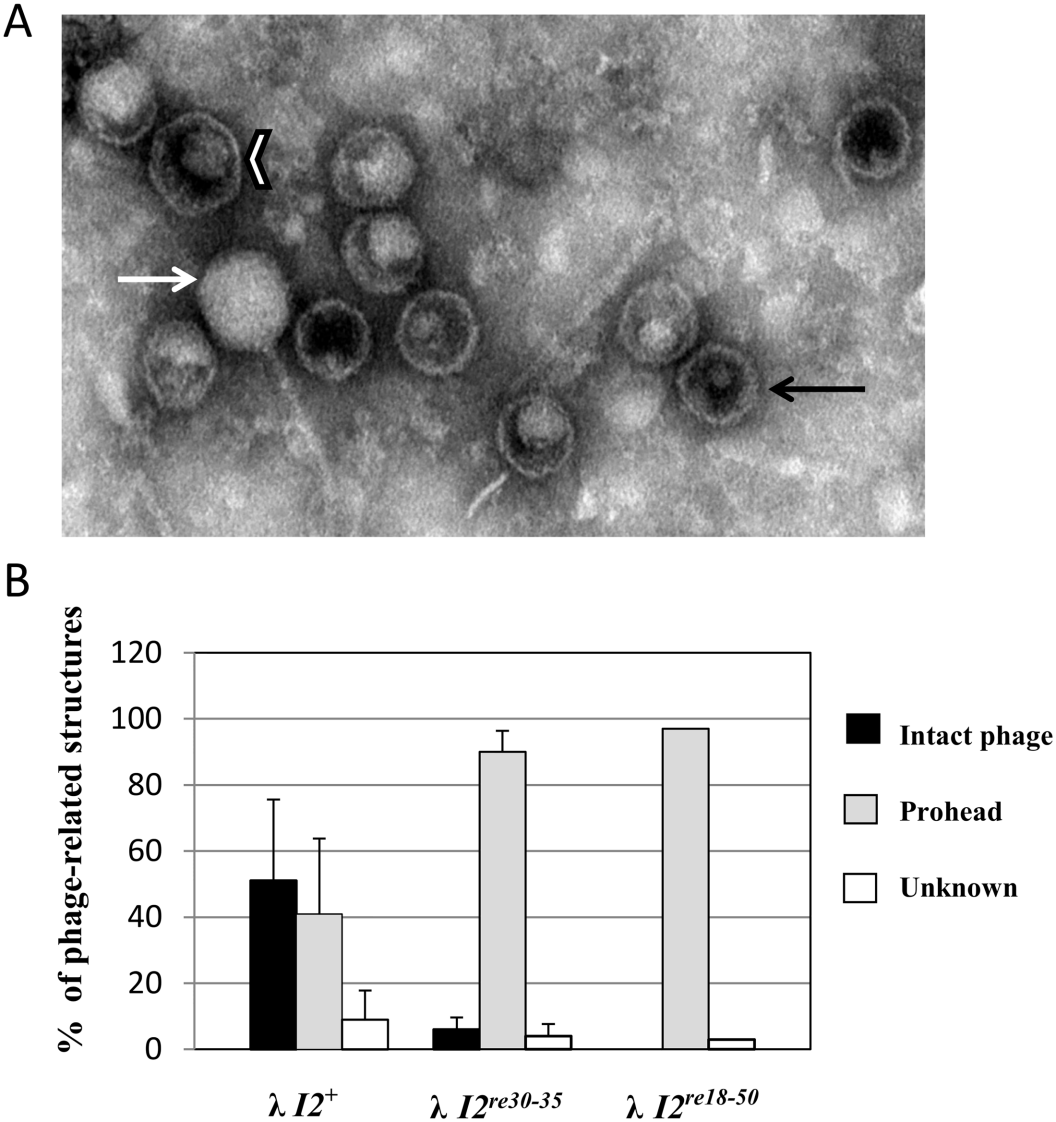
Effects of *I2* mutations on the production of phage-related structures. A. Electron micrograph of phage-related structures in a λ-P1 wild type lysate. Symbols: black arrow = prohead; white arrow = intact phage; white chevron = unknown structure. B. Quantitation of phage-related structures in phage lysates. Total numbers of particles observed were: λ-P1 *I2*^+^—419; λ-P1 *I2*^*re30-35*^–700; and λ-P1 *I2*^*re18-50*^–100. Error bars represent standard deviations of data averaged from several EM preparations. For the *I2*^*re18-50*^ lysate, a single EM preparation was examined.

During DNA translocation, the λ prohead shell undergoes a structural transition to form the mature head shell [41]. The transition is thought to occur when about 30% of the DNA molecule has been translocated [42]. The transition involves an expansion of the shell due to local structural rearrangements by the major capsid protein, gpE [43]. In the electron microscope, the transition is seen as a change from the thick-walled spherical prohead to a larger, more angular and thin-walled form [44]. To ask about the effects of *I2* mutations on phage assembly, we purified phage-related structures from lysates of *I2*^+^, *I2*^*re30-35*^ and *I2*^*re18-50*^. Electron microscopic examination indicated that the *I2* mutants have an early DNA packaging defect, as follows. While lysates of the wild type phage contained roughly equal amounts of proheads and intact phages, the *I2*^*re30-35*^ and *I2*^*re18-50*^ lysates contained nearly all proheads, indicating a DNA packaging defect prior to translocation of 30% of viral chromosome (Fig 4). We next examined initial *cos* cleavage, a packaging step preceding translocation.

### *cos* cleavage *in vivo*

**Fig 5:**
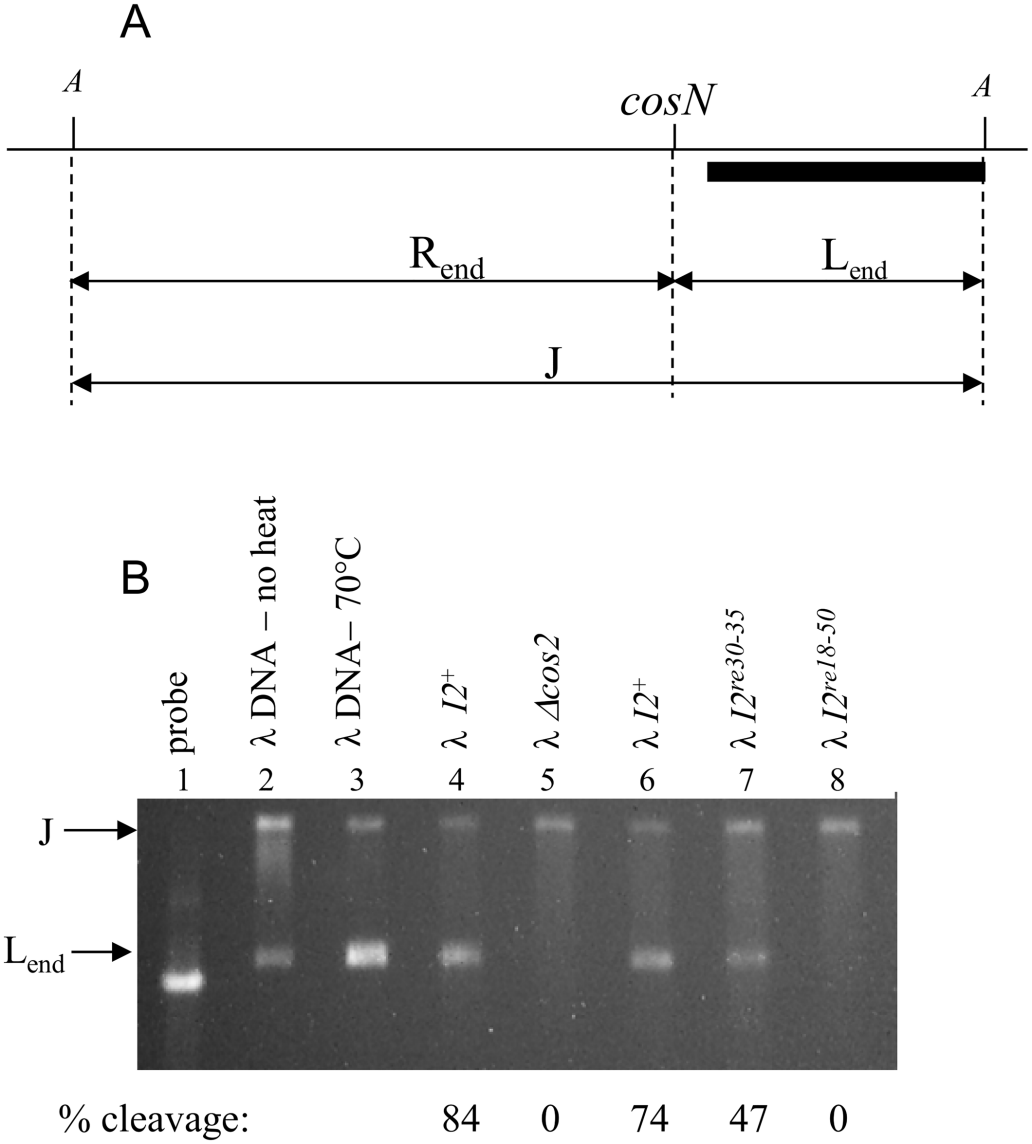
Effects of *I2* mutations on *in vivo cos* cleavage. A. Rationale of the *in vivo cos* cleavage assay. Total phage nucleic acids were isolated from λ-infected cells. AccI digestion of intracellular DNA not cut at *cos* results in a 7681 bp-long joint DNA fragment (J). *cosN* cleavage generates 5591 bp right (R) and 2190 bp left (L) end pieces. In an infection by a mutant that is able to cut *cos* and form complex I, but is unable package DNA, the uncut J and cut L fragments can be detected. AccI-cut total DNA was electrophoresed on a 0.8% agarose gel and transferred to a membrane for southern blotting. B. AccI digested intracellular DNAs: lane 1 is the DNA probe used for Southern blot assay (λ bp 177–2099). Lanes 2 and 3 are AccI-cut λ DNA loaded on the gel before (lane 2) and after heat treatment at 70°C for 10 min to melt the cohesive ends (lane 3). Phage DNAs from positive and negative control phages λ-P1 *I2*^+^ and λ-P1 *cos2* (*ΔcosN*), respectively are in lanes 4 and 5. In lanes 6, 7, and 8 are DNAs from λ-P1 *I2*^+^ (a second sample), λ-P1 *I2*^*re30-35*^ and λ-P1 *I2*^*re18-50*^, respectively.

*In vivo cos* cleavage was studied by examining restriction enzyme (AccI)-digested DNA isolated from induced lysogens. In a wildtype infection, terminase cutting at *cos* cleaves the *cos*-containing joint (J) DNA fragment, producing the nuclease susceptible mature right (R_end_) and the left (L_end_) chromosome end fragments (Fig 5B). The L_end_ DNA is protected from nuclease digestion by assembly into Complex I and by packaging into the prohead. J and L_end_ DNA fragments were detected by Southern blot analysis using a probe homologous to the left chromosome end. The experiment showed that at the time of sampling, about 80% of the DNA had been cut for the *I2*^+^ control phage (Fig 5). For the *I2*^*re30-35*^ mutant, an intermediate level of cutting was observed, and for the *I2*^*re18-50*^ mutant no cleavage was evident. As expected for the λ-P1 *cos2* negative control phage, a *cosN* deletion mutant, no cleavage was found.

### cos cleavage in vitro

**Fig 6:**
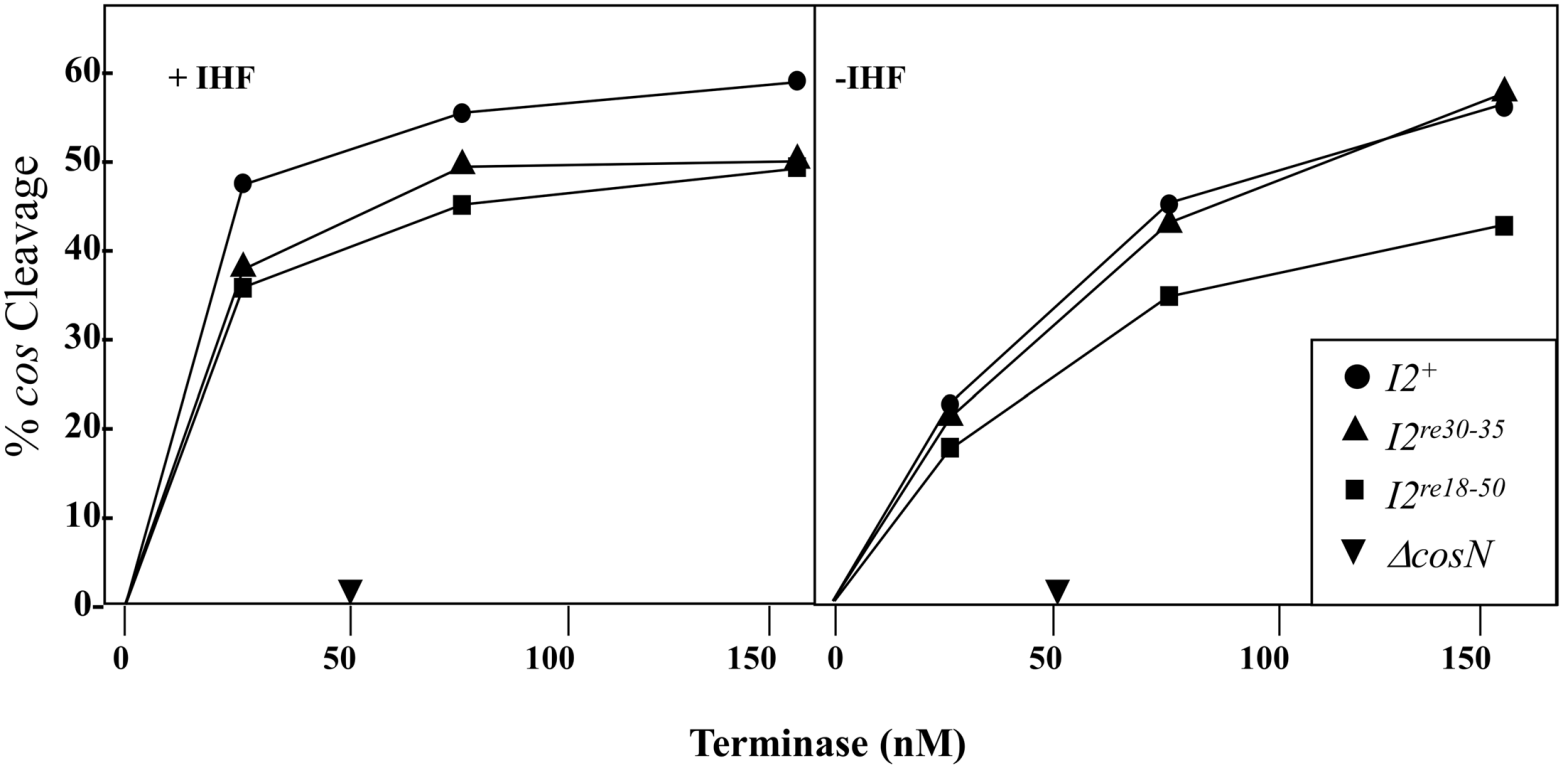
Effect of *I2* mutations on *in vitro cos* cleavage. *I2*-containing 2.9 kb pOER1-5 DNAs (Table 2), linearized with AatII, were used as *cos*-cleavage substrates. After heating at 70°C for 10 min, to melt cohesive ends the 0.6 (L) and 2.3 (R) kb reaction products were run on agarose gels and stained with ethidium bromide. Band intensity was measured with a Typhoon phosphoimager. Reactions were done in the presence (left panel) and absence (right panel) of IHF (see Materials and Methods).

We sought to confirm the *in vivo cos* cleavage results with *in vitro cos* cleavage studies. Linearized cosmid DNA was used as substrate for terminase cleavage reactions that were done in the presence and absence of IHF (Fig 6). With the exception of the *cosN* deletion negative control DNA, each of the substrates, including *I2*^*re18-50*^ and *I2*^*re30-35*^, showed efficient *cos* cleavage. Modest stimulation by IHF was observed at low terminase concentrations, also for all the substrates except the *ΔcosN* DNA used as the negative control. In our experience, *in vitro cos* cleavage results generally correlate well with *in vivo* assays, so the discrepancy between the apparant lack of *cos* clevage *in vivo* and efficient cleavage found *in vitro* is striking.

## Discussion

### *I2* is Critical for a Post-recognition Step in Initiation of DNA packaging

The present results show that, in addition to providing proper spacing between *cosN* and *cosB*, *I2* plays a functional role that is critical for an early step in DNA packaging by λ. The severely lethal *I2*^*re18-50*^ mutation reduces virus yield by ~10^−5^. *I2*^*re18-50*^ is *cis*-acting, preventing the packaging of the mutant DNA but not interfering with DNA packaging by a co-infecting *I2*^+^ phage (Table 1). This *cis*-acting behavior is expected for a site that is involved in a post-recognition interaction required for specific terminase contacts, or perhaps in providing a DNA structure that facilitates contacts with adjacent segments. The *I2* DNA sequence contains poly-dATP tracts, known to create static DNA bending [38]. Gel mobility studies with circularly permuted, *I2*-containing DNA molecules demonstrated that *I2* is bent (Fig 3). Scanning mutagenesis showed that bp 30–35 are critical for *I2* function, and that the flanking segments can be mutated with only mild effects on virus yield (Fig 2). Although lethal, the *I2*^*re30-35*^ mutation reduced virus yield only about ten-fold (Fig 2), and did not greatly affect DNA bending (Fig 3). For the severely defective λ *I2*^*re18-50*^ mutant, lysates contained only unexpanded proheads, indicating that if there is any attempt to translocate DNA, the process fails prior to the prohead expansion event that occurs when about 30% of the DNA has been packaged [42]. For the milder *I2*^*re30-35*^ mutation, reduced levels of expanded proheads and intact phages were observed, indicating *I2*^*re30-35*^ does not completely abolish *I2* function (Fig 4). In *in vitro* reactions, neither I2 mutation had a significant effect on *cos* cleavage, with or without IHF (Fig 5). In contrast, no evidence for *cos* cleavage was found *in vivo* for λ *I2*^*re18-50*^, and an intermediate level of cleavage was found for λ *I2*^*re30-35*^ (Fig 6). The *in vitro cos* cleavage results indicate that *I2* functions at a post-*cos* cleavage step of DNA packaging. Confounding this simple conclusion is the apparent lack of *in vivo cos* cleavage for the severe *I2*^*re18-50*^ mutant, and an intermediate level for λ*I2*^*re30-35*^. These contradictory results are surprising, as previous work on *cos* mutants has generally found a good correlation between *in vitro* and *in vivo* results [19, 20, 45–47]. [Note: a notable exception is that *cos* cleavage *in vivo*, but not *in vitro*, requires assembled proheads and gpFI, as reviewed in [48]. No evidence connects the gpFI/prohead and *I2* discrepancies.] We next consider models for *I2*’s function that account for the *cos* cleavage conundrum.

### Models for *I2*’s function

**Fig 7:**
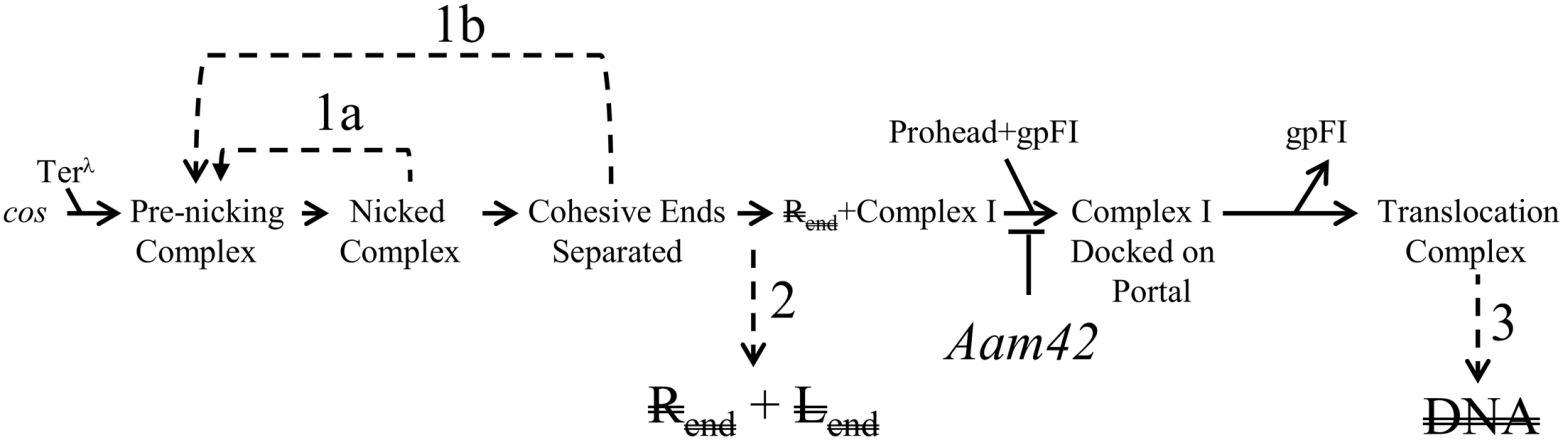
Early DNA packaging steps at which *I2* might act. Four steps at which an *I2* defect might interrupt DNA packaging are numbered. The step blocked for *Aam42* terminase is also indicated. Model 1a: Failure to separate the newly created cohesive ends, followed by dissociation of terminase, and re-ligation that reseals the nicks. Model 1b: Reannealing of the cohesive ends followed by religation. Model 2: *I2* mutations block formation of, or destabilize, Complex I. Note that accompanying Complex I formation, the R_end_ DNA end is released and subject to exonuclease digestion; this is indicated by R_end_. Model 3: *I2* mutations interfere with proper threading of the DNA through the motor assembly so that the DNA is translocated into the cytoplasm and is subject to exonuclease digestion. DNA represents the nuclease-susceptible virion DNA. The *Aam42* defect is the absence of an intact prohead binding domain at the C-terminus of TerL, which prevents Complex I from docking on the portal and assembling an active motor [35, 49, 50].

There is a wide range of possible explanations for the behavior of I2 mutants. To explain the difference between *in vivo* and *in vitro cos* cleavage results, there are possible experimental differences in ionic strength and/or composition, that might account for the observations. Such differences would pertain only to the behavior of the I2 mutants, and not the wild type. For the present discussion, we consider models that invoke known aspects of the λ DNA packaging process. With this constraint, we propose several explanations for the behavior of λ *I2*^*re*^ mutants (Fig 7).

1. An *I2* defect results in cohesive end religation. In this scenario, *cosN* nicking occurs normally for λ *I2*^*re18-50*^, but due to a subsequent defect in the packaging pathway, terminase dissociates from the nicked *cos* and the nicks are resealed by host ligase (Fig 7, Model 1a). This defect might occur if a terminase-*cos* interaction required for strand separation fails to occur. Alternatively, the defect might be in a post-cohesive end separation step at which a required terminase-*I2* interaction is needed to prevent reannealing and religation of the cohesive ends (Fig 7, Model 1b). Models 1a and 1b are testable by directly asking if the intracellular level of the J piece, i.e., the piece with joined cohesive ends of a non-replicating *I2*^*re18-50*^ *cos*, remains constant relative to other phage DNA segments, following late gene expression *in vivo*. In both models, the intracellular level of the J piece is expected to remain constant, even though terminase is active. Models 1a and 1b can be distinguished by looking for a strand separation defect in *in vitro cos* cleavage with the *I2*^*re18-50*^ substrate DNA.

2. An *I2* defect blocks Complex I formation or stability. *I2*^*re18-50*^ might prevent terminase from assembling Complex I, or, if it is assembled, Complex I might be unstable (Fig 7, Model 2). In either case, both newly formed DNA ends would be subject to exonuclease attack. This model presupposes that Complex I is fundamentally different from the nicking and strand separation complexes, and requires critical interactions with the topologically complex *I2*. This scenario, like those of Models 1a and 1b, is testable by quantitating the amount of a non-replicating *I2*^*re18-50*^-containing J piece following late gene expression. If Model 2 is correct, the *I2*^*re18-50*^-containing J piece would decline relative to control segments of the bacterial chromosome. [Note: Attempts by us to demonstrate *in vitro* Complex I assembly with *I2*^+^ and *I2*^*re18-50*^ DNAs were unsuccessful (unpublished).]

3. An *I2* defect derails DNA threading. In this model (Fig 7, Model 3), *I2* mutations do not interfere with Complex I formation or stability, but prevent proper threading of the DNA through the portal so that the DNA is translocated into the cytoplasm and is subject to exonuclease digestion. In this model, the *I2* defect affects translocation, a step that occurs after Complex I formation. This model can be tested by a genetic epistasis test using a previously studied TerL^λ^ mutation, *Aam42*, as follows. The *Aam42* mutation is a chain termination codon such that TerL^*Aam42*^ lacks the C-terminal 5 amino acid residues [35]. *Aam42* terminase is nicking-competent. Solid genetic and biochemical evidence indicates that the C-terminus of wildtype TerL contacts the prohead’s portal vertex [49–51]. During a λ *Aam42* infection, *cos* cleavage is accompanied by formation of Complex I. That is, when intracellular DNA from a λ *Aam42* infection was examined by restriction enzyme digestion and gel electrophoresis, the L_end_ piece, but not the nuclease-susceptible R_end_ piece, was observed [35].

In model 3, an *I2* mutant forms a mal-functioning motor. Model 3 posits that a λ *Aam42 I2*^*re18-50*^ double mutant will have the same phenotype as the *Aam42* single mutant, because the *I2* defect occurs after the *Aam42* defect. Should this genetic test support Model 3, further *in vitro* packaging experiments could ask about misdirected translocation.

Note that each of the models involves a critical terminase-*I2* interaction that fails in the *I2* mutants. The models differ at the point along the DNA packaging pathway at which the crucial interaction occurs. Although the present study provides little structural insight into the nature of the critical interaction, the *I2* mutations provide a clean block to an early packaging step that likely occurs after *cos* cleavage. As little is known about these steps for any DNA virus, the *I2*^*re*^ mutations are valuable tools for dissecting the nature of these steps in the packaging pathway.

### DNA topology in initiation of viral DNA packaging: SPP1 and λ

During progeny virus assembly, virus DNA is specifically selected. In the tailed bacteriophages, recognition involves specific interactions between TerS and the *pac* or *cos* sites. The several TerS structures that have been determined share a three-domain organization [13–15, 52, 53]. The N-terminus is a DNA binding domain (DBD), involved in viral DNA recognition. The DBD is a small α-helical bundle containing a helix-turn-helix (HTH) DNA binding motif. The DBD is tethered to a central domain consisting of two long antiparallel α-helixes that form an antiparallel coiled-coil which further oligomerizes into a hollow cylinder. TerS oligomers contain 8–12 TerS monomers, depending on the virus. At the TerS C-terminus is a β-barrel extension of the cylinder; the C-terminus also contains a specificity domain for interacting with TerL [54, 55]. The DBDs of TerS oligomers are arrayed around the periphery of the central cylinder. A current model proposes that the viral DNA is wrapped around the TerS oligomer to form a nucleosome-like structure [14, 52, 53, 56]. Strong support for nucleosome-like wrapping comes from *pac* phage SPP1, as follows. The SPP1initial cleavage site, *pacC*, is flanked by TerS^SPP1^ binding sites *pacL* and *pacR* sites. TerS^SPP1^ has an extensive footprint at *pac*^*SPP1*^, including *pacL* and *pacR* [56]. *pacL* is intrinsically bent. Footprint experiments strongly indicate that TerS^SPP1^ (G1P) wraps *pacL* into a nucleosome-like structure. TerS^SPP1^ binding is specific, with moderate affinity (K_app_ = 9 nM), and roughly two TerS^SPP1^ oligomers bind *pac*. Thus there is a role for DNA topology at the earliest step, *pacC* cleavage, in SPP1. The pre-cleavage complex for SPP1 possibly has a symmetric feature, as TerS^SPP1^ oligomers bracket *pacC* site. The post-cleavage events leading to docking of SPP1 terminase on the portal vertex are unclear. [Remarkably, the DNA sequences bound by TerS^SPP1^ at *pacL* and *pacR* do not share identity. A recent study indicates that TerS^SPP1^ recognizes local DNA structure rather than a specific sequence [57].]

λ’s pre-cleavage nucleoprotein assemblage also involves DNA topology, in this case the IHF-enhanced intrinsic bend at I1. In contrast to SPP1, the TerS^λ^ assemblage at *cosB* is asymmetric, being located on only one side of *cosN*. The two-fold rotational symmetry of *cosN* suggests that cleavage is carried out by symmetrically disposed TerL^λ^ monomers. I2’s static bend acts at a second, post-cleavage step of packaging, as discussed above. Whether there is a similar role for DNA topology in a post-cleavage packaging step for SPP1 and other DNA viruses remains to be learned.

### Post-cleavage Gymnastics

The assembled translocation motor includes a pentameric TerL ring, in both T4 [58] and Phi29 [59, 60]). In both cases, the TerL molecules are asymmeteically arrayed on the prohead’s portal protein. Especially in the case of λ, the transition from the pre-nicking complex with symmetrically disposed TerLs, to the translocation motor, with asymmetrically arrayed TerLs, suggests there might be a substantial rearrangement of TerLs during the transition. This line of thought contrasts with biochemical studies suggesting that TerS_2_:TerL_1_ protomers assemble tetramers; the resulting tetramers are fully competent for both *cos* cleavage and translocation [16, 17]. Electron microscopic examination indicates that the tetramers possess great structural plasticity [61]. It is thus possible that the tetramer’s endonuclease domains could adopt both symmetric and asymmetric orientations without major rearrangement of the global structure of the tetramer.

The final point in the DNA packaging cycle where a rearrangement is indicated is termination. In λ, cutting of the downstream *cos*, to finish DNA packaging, requires *cosQ* [62]. In the absence of *cosQ*, translocation does not arrest at the downstream *cos*, rather packaging continues, resulting in failure to terminate the chromosome being packaged, a lethal event. Examination of the packed downstream *cos* shows that the top DNA strand is properly nicked but the bottom strand is not nicked [27]. It is argued that *cosQ* acts to present a symmetrically disposed TerL endonuclease domain to *cosN*’s bottom strand. Again, the role of *cosQ* might include a major rearrangement of terminase architecture, or a local repositioning of an endonuclease domain. A third possibility is that *cosQ* recruits a new terminase protomer to enable *cosN* cleavage [27]. The one clear conclusion is that much remains to understand about terminase architecture and dynamics during DNA packaging.

## Materials and Methods

### Strains and Media

Luria broth (LB), LB agar, tryptone broth (TB), tryptone broth agar (TA), and tryptone broth soft agar (TBSA) were prepared as described [63], except TB, TA, and TBSA were supplemented with 0.01 M MgSO_4_. For phage infections, TB was supplemented with 0.2% maltose. When required, ampicillin, chloramphenicol, and kanamycin were added at 100 μg/mL, 10 μg/ml, and 50 μg/mL, respectively.

Bacteria, phages and plasmids are listed in Table 2.

**Table 2:** Bacteria, Phages, and Plasmids.

Strain/Plasmid	Genotype/Comment/Source or Reference
A. Bacteria	
MF3510	W3101 *recA1 galK103* / Lab collection
MF1427	C1a *galK* / [64]
C600	*thi leu thr supE* / host for plaque assays / Lab collection
C600 (λ^+^)	*thi leu thr supE* / recipient for kanamycin transduction assays / Lab collection
MF1419	*thyA met nadBF ung-1 gal supE supF hsdR*^-^*hsdM*^+^/ [65]
B. Phages	
λ-P1	λ-P1:5R Kn^R^ *cI857 nin5* [66, 67]/ Wild type for *cos*^*λ*^ *terS*^*λ*^ *terL*^*λ*^. Forms plasmid prophage. Mutations in mutant derivatives: *cos2* [68], *Sam7* [69] and *Aam42* [35]
λ-P1 *I2*_i_	Derivatives of λ-P1 carrying *I2* replacement mutations: *I2*^*re18-50*^, *I2*^*re18-29*^, *I2*^*re30-35*^, *I2*^*re36-41*^ and *I2*^*re42-47*^ / This work
λ*cI857 red3 att*^+^	Wild type for *cos*^*λ*^ including *I2*^+^, *terS*^*λ*^ *terL*^*λ*^. Prophage is integrated in bacterial chromosome.
C. Plasmids	
pBUC8	*I2*^+^ cosmid: pUC19 carrying λ DNA from SapI site at 47712 to MluI site at 458. / Lab collection
pBC2	*I2*^*re18-50*^ cosmid derivative of pBUC8This work
pOER1	*I2*^+^ cosmid. pUC119 is a derivative of pUC19 lacking the multicloning site. λ DNA insert extends from 48442 to 178. / Blue Heron Biotechnology Inc.
pOER2, 3,4, and 5	Derivatives of pOER1 carrying *I2*^*re18-29*^, *I2*^*re30-35*^, *I2*^*36-41*^ and *I2*^*42-47*^, resp. / This work
pBend	Plasmid for circular permutation mobility assays / [40]
pBend derivatives	*I1*^+^, *I2*^+^, *I2*^*re30-35*^, *I2*^*re18-50*^: 35 or 36 bp segments cloned into the XbaI and SalI sites of pBend; sequences are in S1 Table. / This work

### Induction of lysogens

In general, the following procedure was used to make lysates for the cosmid packaging assay, burst size determination, phage x plasmid crosses, and *in vivo cos* cleavage assay. Lysogens were grown in 5 ml of LB at 31°C to a cell density of ~5 x 10^7^ cells per ml, transferred to 42°C for 20 min for prophage induction, and then aerated at 37°C for a 70 min phage growth period. After CHCl_3_ was added to lyse the cells, the lysate was clarified by centrifugation in a clinical centrifuge for 10 min at 4°C. To obtain counts of induced cells, dilutions of the cultures were made before induction at 42°C and plated at 30°C. Lysates were diluted in 10 mM MgSO_4_ when required for titrations.

### Plasmid constructions

To construct pBUC8 and pBC2, a 1248 bp fragment (SapI, bp 47,712 to MluI, bp 458) of λ DNA was cloned into pUC19 using standard methods, to create pBUC8. The sequence corresponding to λ bp 18–50 in pBUC8 was replaced with scrambled sequence to make the analogous plasmid, pBC2, containing the *I2*^*re18-50*^ mutation, as follows. The middle of the *I2*^*re18-50*^ mutant sequence from bp 30 to 35 is a recognition site for the PstI restriction enzyme. Oligonucleotides with the left and right halves of the *I2*^*re18-50*^ mutation, including the PstI sequence, were used in PCR amplifications to produce XbaI (48442) to PstI (30) and PstI (30) to MluI (458) segments that were cloned into pUC19. The left and right half *I2*^*re18-50*^ segments were subsequently ligated at the PstI site to produce the *I2*^*re18-50*^ containing Xba-to-Mlu segment, which was used to replace the *I2*^+^ sequences in pBC2 (Table 2). Plasmids pOER1, pOER2, pOER3, pOER4, and pOER5 have a 238 bp insert (λ bp 48446–182) that includes *cosQ*, *cosN*, and *cosB*, flanked by XbaI and XmaI restriction sites at the 5’ and 3’ ends, respectively. The pOER series of plasmids was purchased from Blue Heron (http://www.blueheronbio.com, Bothell, WA). The plasmid vector was pUC119, a derivative of the standard vector pUC19.

### *In vivo* cosmid packaging

Plasmids pBUC8 and pBC2 were used to transform MF1427(λ-P1:5R *cI857 nin*5 *Sam7* Kn^R^) to Ap^R^ by standard methods [70]. Lysates of MF1427 (λ-P1:5R *cI857 nin5 Sam7* Kn^R^)[pBC2] and MF1427(λ-P1:5R *cI857 nin5 Sam7* Kn^R^)[pBUC8] were prepared as described in “Induction of lysogens”, this section. Titers of ampicillin resistance transducing particles were determined by mixing 100 μl aliquots of lysate dilutions with 200 μl of overnight cultures of MF1427(λ^+^), followed by a one hour incubation at 31°C, and then plating dilutions on LB plus ampicillin plates and incubating at 31°C.

### Assay for *cis*-specificity: Dilysogen construction and burst size determinations

In this experiment, the λ *I2*^+^ prophage was *att*^+^ *gam*^+^ *red3* and resided in the host chromosome. The λ *I2*^*re18-50*^ prophage background was λ-P1, which forms a plasmid prophage. Because λ-P1’s Kn^R^ cassette is inserted between the Sal sites at λ bps 32745 and 33244, the phage is Δ(*gam-bet*) and is defective in generating concatemeric, packageable DNA in a *recA* host, such as used here. The *bet* defect abolishes Red recombination, one source of concatemers, and the *gam* defect permits RecBCD nuclease attack of rolling circle replication, another source of concatemers [71]. In addition, the defect in producing concatemers results in a reduced late gene dosage, reducing the level of proteins involved in virion assembly. In the complementation experiment, λ *I2*^+^ provides Gam, permitting rolling circle replication by λ-P1 *I2*^*re18-50*^.

### Crossing *I2* mutations into phage

Plasmids pOER1, pOER2, pOER3, pOER4, and pOER5 were used to transform MF1427(λ-P1:5R *cI857 nin*5 *Sam7 cos2* Kn^R^) to ampicillin resistance by standard methods. Plasmid versus phage crosses were carried out by prophage induction, and the resulting cross lysates were examined for plaque forming recombinants by plating on MF1427 in TBSA at 37°C. Plaque forming recombinants were examined for *I2* markers by sequencing appropriate PCR products. The inviable *I2*^*re30-35*^ recombinant was isolated by screening kanamycin resistant transductants of MF1427 for presence of a prophage unable to release plaque forming phages, followed by sequencing PCR products.

### Determination of static bend

Permutation analysis was done with a 150 bp DNA fragment containing *I2*^+^, *I2*^*re30-35*^, or *I2*^*re18-50*^ sequences, along with *I1*^+^ as a positive control. (A) A 35 bp DNA fragment containing λ *I2*^+^, *I2*^*re30-35*^, *I2*^*re18-50*^, or *I1*^+^ sequence was inserted into the XbaI and SalI sites of the vector pBend2 [40]. When a restriction enzyme cuts a site in the tandem repeat segment that flanks the insert, a 150 bp DNA fragment is produced which contains the *I2* insert at various positions (Fig 3A). These permuted fragments were electrophoresed in an 8% polyacrylamide gel in 1x TBE buffer (150 mM Tris, 32 mM boric acid, 1 mM EDTA, pH 8.3) for 19 hours at 150 V at 4°C. The gel was stained in ethidium bromide (1 μg/ml) for 15 min, washed in 1x TBE buffer, and then visualized under UV light. (B) Relative sizes of the permuted DNA fragments in (A) are plotted against the positions of the restriction site (permutation position). A relative mobility of 1.0 indicates the mobility of a 150 bp DNA marker. The DNA bending angles (α) are adjacent to the appropriate mobility plot. The bend angle for each permuted fragment was calculated by following an empirical relationship between the relative electrophoretic mobility retardation caused by bending and the bending angle [40].

### Electron Microscopy

Pelleted phages were resuspended and applied to 400 mesh copper grids coated with Formvar and carbon. The grids were then negatively stained with 2% potassium phosphotungstate (pH 6.5) for 1 min, and examined under transmission electron microscope at 30,000X.

### *In vitro cos* cleavage

20 μl reactions containing 10 mM pUC119 DNA having a 234 bp *I2*^+^, *I2*^*re30-35*^, *I2*^*re18-50*^, or *cos2* insert, and purified terminase were done as reported previously [72]. Terminase concentration was calculated using the A_280_ and the extinction coefficient of ε = 157.2 mM^-1^cm^-1^ [73]. Reaction products were run on a 0.8% agarose gel and stained with ethidium bromide. The inserts contain the *cosN* cutting site. Terminase concentrations varied from 0 to 150 nM. The percent DNA cut at *cos* was determined by analysis of product bands with a computer imaging program. Product band intensity was measured on a Typhoon-8600 phosphoimager (Molecular Dynamics). Parallel reactions with 50 nM purified IHF, kindly given by Helene Gaussier and Carlos Catalano, were also analyzed.

### *In vivo cos* cleavage assay

The *in vivo cos* cleavage assay was performed as described in “Induction of lysogens”, this section [74]. Total DNA was isolated by extractions with phenol:chloroform:isoamyl once, with phenol two times, and then with chloroform once, followed by ethanol precipitation. DNA pellets were air-dried and then resuspended in 40 μl of 10 mM Tris. DNA (10 μl) was cut with AccI for 2 h at 37°C, heated at 70°C for 10 min to melt cohesive ends, applied to a 0.8% agarose for gel electrophoresis, and then transferred to GeneScreen Plus (New England Nuclear) membrane using a vacuum blotting transfer system (American Bionics). A PCR fragment of λ bp 177 to 2099 was labeled with DIG-11-dUTP (Roche) according to the supplier’s instructions and then used to probe the L_end_ (λ bp 1 to 2190) and J (λ bp 42921 to 2190) AccI fragments. Bands were detected by chemiluminescence (Pierce). The percent *cos* cleavage was calculated using phosphoimaging data, according to the following formula: (L_end_ x 100)/(L_end_ + J), where L_end_ and J equal L_end_ and J fragment brightness, resp. Brightness values were corrected for background. Phosphoimaging was done using the Image Reader LAS-1000 of the Typhoon 8600 photoimager (Molecular Dynamics).

## Supporting Information

S1 Table Oligonucleotides used as pBend Inserts. (DOCX)
