# Supplementary material for: DNA Topology and the Initiation of Virus DNA Packaging
Source: PLoS One. 2016 May 4;11(5):e0154785. doi: 10.1371/journal.pone.0154785 (PMC4856287; doi:10.1371/journal.pone.0154785)
Supplement: S1 Table — (DOCX) [file pone.0154785.s001.docx]

S1Table: Oligonucleotides used as pBend Inserts

| DNA segment | pBend Inserts |
| --- | --- |
| *I1^+^*  (λ bp 65-100) | 5’-ctagaTCTTCGTCATAACTTAATGTTTTTATTTAAAATACCg-3’  3'-tAGAAGCAGTATTGAATTACAAAAATAAATTTTATGGcagct-5’ |
| *I2^+^*  (λ bp 18-52) | 5’-ctagaGTTTTCGCTATTTATGAAAATTTTCCGGTTTAAGG-3’  3'-tCAAAAGCGATAAATACTTTTAAAAGGCCAAATTCCagct-5' |
| *I2^re18-50^*  (λ bp 18-52) | 5’-ctagaCAGAGTCTCTAGCTGCAGTCAGACGATACAGTCGG-3’  3’-tGTCTCAGAGATCGACGTCAGTCTGCTATGTCAGCCagct-5' |
| *I2^re30-35^*  (λ bp 18-52) | 5’-ctagaGTTTTCGCTATTGGGCCCAATTTTCCGGTTTAAGG-3’  3'-tCAAAAGCGATAACCCGGGTTAAAAGGCCAAATTCCagct-5' |

λ sequences are underlined, replacement mutations are highlighted in magenta.
